# Innovative retargeted oncolytic herpesvirus against nectin4-positive cancers

**DOI:** 10.3389/fmolb.2023.1149973

**Published:** 2023-05-11

**Authors:** Andrea Vannini, Federico Parenti, Cristina Forghieri, Catia Barboni, Anna Zaghini, Gabriella Campadelli-Fiume, Tatiana Gianni

**Affiliations:** ^1^ Department of Medical and Surgical Sciences (DIMEC), University of Bologna, Bologna, Italy; ^2^ Department of Pharmacy and Biotechnology, University of Bologna, Bologna, Italy; ^3^ Department of Veterinary Medical Sciences, University of Bologna, Bologna, Italy

**Keywords:** oncolytic virus, oncolytic herpes simplex virus, retargeting, nectin4, anti-cancer vaccine, immunotherapy, pancreas carcinoma, triple negative breast cancer

## Abstract

Nectin4 is a recently discovered tumor associated antigen expressed in cancers that constitute relevant unmet clinical needs, including the undruggable triple negative breast cancer, pancreatic ductal carcinoma, bladder/urothelial cancer, cervical cancer, lung carcinoma and melanoma. So far, only one nectin4-specific drug—Enfortumab Vedotin—has been approved and the clinical trials that test novel therapeutics are only five. Here we engineered R-421, an innovative retargeted onco-immunotherapeutic herpesvirus highly specific for nectin4 and unable to infect through the natural herpes receptors, nectin1 or herpesvirus entry mediator. *In vitro*, R-421 infected and killed human nectin4-positive malignant cells and spared normal cells, e.g., human fibroblasts. Importantly from a safety viewpoint, R-421 failed to infect malignant cells that do not harbor nectin4 gene amplification/overexpression, whose expression level was moderate-to-low. In essence, there was a net threshold value below which cells were spared from infection, irrespective of whether they were malignant or normal; the only cells that R-421 targeted were the malignant overexpressing ones. *In vivo*, R-421 decreased or abolished the growth of murine tumors made transgenic for human nectin4 and conferred sensitivity to immune checkpoint inhibitors in combination therapies. Its efficacy was augmented by the cyclophosphamide immunomodulator and decreased by depletion of CD8-positive lymphocytes, arguing that it was in part T cell-mediated. R-421 elicited *in-situ* vaccination that protected from distant challenge tumors. This study provides proof-of-principle specificity and efficacy data justifying nectin4-retargeted onco-immunotherapeutic herpesvirus as an innovative approach against a number of difficult-to-drug clinical indications.

## 1 Introduction

Oncolytic virotherapy is being developed as an innovative approach to cancer therapy (Martuza et al., 1991; Cattaneo and Russell, 2017; Russell and Peng, 2017). It entails the use of viruses–either natural strains or genetically engineered recombinants–that selectively infect, replicate in and destroy cancer cells, and spare non-malignant cells. Interest in oncolytic viruses (OVs) greatly increased with the recognition that they exert anticancer activity by enhancing the host’s immune response to cancer cells, thanks to the induction of immunogenic cell death and subversion of the immunosuppression typical of tumor microenvironment (TME). As a result, they confer, or anyhow increase, sensitivity to immune checkpoint inhibitors (ICIs) (Keller and Bell, 2016; Senior, 2019). As of today, OVs constitute a branch of the immunotherapy of cancer (oncoimmunotherapeutic viruses, OIVs) (Topalian et al., 2011; Lichty et al., 2014; Bommareddy et al., 2018; Senior, 2019). The wealth of preclinical and clinical studies culminated in the approval of two herpesviruses for clinical applications, namely, the Food And Drug Administration (FDA) and European Medicines Agency (EMA) approval of the OncoVEX^GM−CSF^, also named Talimogene laherparepvec (T-VEC) or Imlygic, for the therapy of cutaneous melanoma and, in Japan, the approval of Teserpaturev, also named G47∆ or Delytact for the therapy of malignant gliomas (Hughes et al., 2014; Andtbacka et al., 2015; Todo et al., 2022). The adenovirus Oncorine has been approved in China against head and neck cancer (Liang, 2018). Given the intensity of ongoing preclinical and clinical studies, it is expected that more OVs will be approved in the near future.

Tropism-retargeted OVs, including the tropism-retargeted herpesviruses (ReHVs) developed in our laboratory, represent a distinct group of these agents (Menotti et al., 2008; Menotti et al., 2009; Campadelli-Fiume et al., 2011; Nanni et al., 2013). Entry of the wild-type (wt) herpes simplex virus type 1 (HSV-1) into the cells requires the coordinated activity of four essential glycoproteins, activated in a cascade fashion (Campadelli-Fiume et al., 2012). The receptor-binding glycoprotein D (gD) interacts with the major natural receptors nectin1 or HVEM (herpesvirus entry mediator) (Cocchi et al., 1998b; Warner et al., 1998), activates the intermediate gH/gL heterodimer that, upon release of gL, activates the executor of fusion gB (Heldwein et al., 2006; Gianni et al., 2015; Campadelli-Fiume et al., 2016a). Full retargeting entails two sets of modifications, namely, the detargeting from natural receptors by genetic modifications of gD and the retargeting by insertion of a novel ligand in gD, gH, or gB, or combinations thereof (Gatta et al., 2015; Campadelli-Fiume et al., 2016b; Leoni et al., 2017; Petrovic et al., 2017; Leoni et al., 2018a; Petrovic et al., 2018). The novel ligand targets a tumor associated antigen (TAA) of choice, i.e., a molecule selectively expressed on the surface of cancer cells and not expressed or expressed at moderate-to-low levels in normal cells. Each ReHV specifically targets the set of indications that share the same TAA and thus fall into the personalized medicine approach. In contrast to the majority of oncolytic herpesviruses that gain cancer-selectivity and safety by varying degrees of attenuation, mainly by deletion/mutation of the γ34.5 gene (Martuza et al., 1991; Chambers et al., 1995; Liu et al., 2003; Vannini et al., 2021b), ReHVs are cancer-specific agents, exhibit a high safety profile in murine models, carry no attenuating deletion/genetic modification, and are γ34.5-positive. So far, our laboratory has engineered and preclinically tested ReHVs retargeted to HER-2 (epidermal growth factor receptor 2), EGFR (epithelial growth factor receptor), EGFRvIII, PSMA (prostate specific membrane antigen) (Menotti et al., 2008; Menotti et al., 2009; Menotti et al., 2018; Vannini et al., 2021c). Inasmuch as ReHVs are virulent viruses within the targeted malignant cells, they elicit a strong innate and adaptive immune response and *in situ* anti-cancer vaccination. The vaccination effect is augmented by the engineering in the ReHV genome of adjuvant molecules, such as IL-12 (interleukin-12), or GM-CSF (granulocyte-macrophage colony-stimulating factor), etc., (Liu et al., 2003; Hughes et al., 2014; Leoni et al., 2018b; De Lucia et al., 2020; Vannini et al., 2021a).

TAAs are drug targets. In clinical practice, they are targeted by a number of therapeutics, mainly monoclonal antibodies, their drug-conjugated derivatives, and small molecule inhibitors. The necessity to develop new therapeutics to cancers that represent unmet clinical needs fosters the search for previously unknown TAAs. Nectin4 was discovered as an adhesion molecule belonging to the nectin family of the immunoglobulin superfamily (Reymond et al., 2001), and was then recognized as being highly expressed in clinically relevant cancer indications, including bladder and urothelial cancers, pancreatic carcinoma, some breast cancers, including the undruggable triple negative breast cancer, cervical cancer, lung carcinomas and, recently, melanoma (Deng et al., 2019; Chatterjee et al., 2021; Heath and Rosenberg, 2021; Tanaka et al., 2021; Bouleftour et al., 2022; Hashimoto et al., 2022). It is being explored as serum marker (Fabre-Lafay et al., 2005; M-Rabet et al., 2017; Sanders et al., 2022). As is the case for almost any TAA, nectin4 is either not expressed in most normal tissues (Reymond et al., 2001; Deng et al., 2019), or is moderately-to-lowly expressed in some tissues, including skin, esophagus, bladder, salivary glands, female tissues, etc., (Uhlén et al., 2015). The development of nectin4–specific drugs is in its infancy. Enfortumab Vedotin (commercial name Padcev) is a drug-conjugated human monoclonal antibody to nectin4, and the first-in-class nectin4-specific drug (Challita-Eid et al., 2016; Hoffman-Censits et al., 2021; Powles et al., 2021). It was initially approved by the FDA at the end of 2019 as third-line agent against advanced bladder and urothelial carcinomas resistant to ICIs and is being evaluated as first-line agent in combination with pembrolizumab. It is currently approved for locally advanced or metastatic urothelial cancer. Other agents are in preclinical or very early clinical stages of development. Indeed, very few (five) clinical trials are ongoing that test Enfortumab Vedotin, other drugs, or CAR-Ts against nectin4-positive indications. The use of Enfortumab Vedotin is associated with adverse effects, mainly cutaneous toxicities that can be fatal and with drug-resistance (Wu and Adamson, 2019; Dobry et al., 2021; Francis et al., 2021; Cabaud et al., 2022; Enescu et al., 2022; Guerrois et al., 2022; Klümper et al., 2022).

Proof of principle evidence was provided that nectin4-positive cancers can be targeted by an oncolytic measles virus in immunodeficient murine models. Immunotherapy, long-lasting protection, and the combination with ICIs were not addressed (Amagai et al., 2016; Awano et al., 2016; Fujiyuki et al., 2020).

The objectives of current study were to ascertain whether *in vitro* human cancer cell lines expressing nectin4 were susceptible to and killed by a nectin4-retargeted onco-immunotherapeutic herpesvirus (nectin4-ReHV), whether susceptibility was influenced by the extent of nectin4 expression, to develop immunocompetent murine models of human nectin4-positive cancers, and to evaluate mono- and combination therapy and long-term anticancer vaccination.

## 2 Materials and methods

### 2.1 Cells

MDA-MB-468 (triple negative human breast adenocarcinoma), MDA-MB-231 (triple negative human breast adenocarcinoma), MCF-7 (human breast adenocarcinoma), SK-BR-3 (human breast adenocarcinoma), A-431 (human epidermoid carcinoma), BxPC-3 (human pancreatic adenocarcinoma), CAPAN-1 (human pancreatic adenocarcinoma), PANC-1 (human epithelioid carcinoma of pancreas), HPAC (human pancreatic adenocarcinoma), SK-OV-3 (human ovarian adenocarcinoma), LLC1 (mouse Lewis Lung carcinoma), CT26 (mouse colon carcinoma), and Renca cells (mouse renal adenocarcinoma), U-251 (human glioblastoma) U-2-OS (human epithelial osteosarcoma), MRC-5 (normal human fibroblast), HeLa (human epithelial adenocarcinoma) were purchased from ATCC (Manassas, VA, United States) and cultured as indicated by ATCC. HFF cells (primary human foreskin fibroblasts) were provided by Frank Neipel, University of Erlangen, J cells (a derivative of BHK-TK− cells lacking any HSV receptor and resistant to infection with HSV) (Cocchi et al., 1998a) and their derivatives expressing the natural receptors nectin1 or HVEM, or expressing human nectin4 were grown in DMEM (#31600-083, Gibco Laboratories) supplemented with 5% fetal bovine serum (FBS). K-562 (human lymphoblast chronic myelogenous leukemia) were kindly provide by Scott S. Blystone, Upstate Medical University, Syracuse and cultured in Iscove’s modified Dulbecco’s medium supplemented with 10% FBS.

### 2.2 Determination of nectin4 expression and generation of murine and human cancer cells expressing human-nectin4

Cell surface expression of nectin4 in human malignant (MDA-MB-468, MCF-7, SK-BR-3, A-431, BxPC-3, CAPAN-1, SK-OV-3, MDA-MB-231, PANC-1, HPAC, U-251, U-2-OS, HeLa, K-562) and non-malignant (HFF, MRC-5) cells was determined by flow cytometry. Single-cell suspensions were incubated with polyclonal antibody (PAb) to nectin4 (Invitrogen, PA5-30837; diluted 1:100) for 1 h in ice, washed 3 times with FACS buffer (PBS + 2% FBS), incubated with anti-rabbit Alexa Fluor 488-conjugate secondary antibody (Invitrogen, A11070) for 1 h in ice, and rinsed with FACS buffer. Fluorescence was determined by flow cytometry (BD Accuri). SK-OV-3-wt, J-wt, LLC1-wt, CT26-wt, and Renca-wt cells were made transgenic for the expression of human nectin4 (hN4). Briefly, the human nectin4 (GenScript, Clone ID: Ohu28639D in pcDNA3.1+/C-(K)-DYK) plasmid was amplified with primers Forw_N4_BamHI (TAC​TAC​TTg​gat​cAT​GTA​TCC​TTA​CGA​CG) and Rev_N4_XhoI (AAG​TAG​TAc​tcg​agT​TAG​ACC​AGG​TGT​CC). The BamHI-XhoI digested PCR fragment was cloned into the lentiviral expression vector pLV-EF1-MCS-SV40-Puro, obtaining pLV-Nect4-puro. Cells were transduced as previously detailed (Leoni et al., 2018a). Transduced cells (named SK-OV-3-hN4, J-hN4, LLC1-hN4, CT26-hN4, and Renca-hN4, respectively) were selected by means of puromycin. Single cell clones were obtained by limiting dilution. Clones were checked for stable nectin4 expression for up to 40 passages in cell culture by flow cytometry with PAb to human nectin4.

### 2.3 Viruses

The R-LM5 virus was described (Menotti et al., 2008). To obtain the R-421 recombinant virus, the gD gene in BAC-337 (Vannini et al., 2021b) was modified by galK recombineering such that the scFv to HER2 was replaced with the scFv directed against nectin4 (Menotti et al., 2020; Vannini et al., 2020). Briefly, the *galK* cassette with homology arms to gD was amplified with primers gD37GalKFor ACC​TTC​CGG​TCC​TGG​ACC​AGC​TGA​CCC​CTC​CGG​GGG​TCC​GGC​GCG​TGC​CTG​TTG​ACA​ATT​AAT​CAT​CGG​CA and gD39GalKRev ATC​GGG​AGG​CTG​GGG​GGC​TGG​AAC​GGG​TCT​GGT​AGG​CCC​GCC​TGG​ATG​TGT​CAG​CAC​TGT​CCT​GCT​CCT​T, using pGalK as template, and the amplicon was employed for the first step of recombineering. The plasmid pCDNA3.1(+)-Nect4-VLVH coding for the scFv to nectin4 (Geneart, Thermofisher Scientific) was designed with VL (DIVL … LEIK, 111 aa) and VH (EVLL … TVSA, 115 aa) sequences (Lopez, 2018) linked by means of the gD linker sequence SDMPMADPNRFRGKNLVFHS; it harbored a SSGGGSGSGGSG linker downstream of the VH portion. The cassette of scFv to nectin4 with homology arms to gD was amplified with primers nect_gD_for ACC​TTC​CGG​TCC​TGG​ACC​AGC​TGA​CCC​CTC​CGG​GGG​TCC​GGC​GCG​TGG​ACA​TCG​TGC​TGA​CAC​AGA​GC and nect_gD_rev ATC​GGG​AGG​CTG​GGG​GGC​TGG​AAC​GGG​TCT​GGT​AGG​CCC​GCC​TGG​ATG​TGA​GAT​CCT​CCG​CTT​CCA​GAT​C, using pCDNA3.1(+)-Nect4-VLVH as template, and the amplicon was employed for the second step of recombineering. R-421 virus was reconstituted in SK-OV-3-hN4 cells, single plaque purification was performed (Menotti et al., 2020; Vannini et al., 2020), and viral DNA was checked for the excision of BAC sequences (Vannini et al., 2021c). R-421 virus was cultivated in SK-OV-3-hN4 cells.

### 2.4 R-421 tropism and extent of infection

J-hN4, SK-OV-3-hN4, human cancer cells expressing human nectin4 at high levels (hN4-high) (MDA-MB-468, MCF-7, SK-BR-3, A-431, BxPC-3, CAPAN-1), or at moderate-to-low levels (hN4-low) (MDA-MB-231, PANC-1, HPAC, U-251, HeLa, K-562), hN4-low non-malignant cells (HFF, MRC-5) and the murine LLC1-hN4, CT26-hN4, Renca-hN4 cells were infected with R-421 at an input multiplicity of infection (MOI) of 10 PFU/cell as titrated in SK-OV-3-hN4 cells. Absorption was for 90 min at 37°C. Infection was monitored 24 h later as the number of EGFP expressing cells by flow cytometry, or as pictures taken at Nikon Eclipse TS100 fluorescence microscope. All fluorescence images were taken with the same exposure time of 800 m-sec. Comparison of R-421 and R-337 infection in SK-OV-hN4 cells (input MOI 10 PFU/cell) was performed by indirect immunofluorescence assay (IFA) at 24 h after infection (Gianni et al., 2021). Briefly, methanol-fixed cells were incubated with MAb BD80 (Petrovic et al., 2017) to HSV-1 gD (1:100 dilution), rinsed and incubated with anti-mouse Alexa Fluor 488-conjugated secondary antibody (Invitrogen, A11001) (1:200 dilution). Western blot assay with MAb BD80 was performed as described (Gianni et al., 2012).

### 2.5 Efficiency of infection, virus growth, plaque formation and cytotoxicity assay

To determine the efficiency of R-421 infection across different cell lines, serial dilutions of a fixed amount of virus were plated on the indicated cell lines. Infected cultures were overlaid with medium containing agar. Plaque numbers were scored 5 days later. The relative efficiency of R-421 infection in any given cell line (also referred to as plating efficiency in different cells) was expressed as the percentage of the number of plaques formed by the fixed amount of R-421 relative to the number of plaques formed by the same amount of virus in SK-OV-3-hN4 cells, referred to as 100%. For plaque size determinations, pictures of 10 independent plaques were taken for each cell line. Plaque areas were measured with Nis Elements-Imaging Software (Nikon). To quantify the extent of virus replication, hN4-high cells (MDA-MB-468, MCF-7, SK-BR-3, A-431, BxPC-3, CAPAN-1, SK-OV-3-hN4, LLC1-hN4, CT26-hN4, Renca-hN4) were infected at an input multiplicity of 0.1 PFU/cell, according to the titer determined in the same cell line. Unabsorbed virus was inactivated by means of acid wash (40 mM citric acid, 10 mM KCl, 135 mM NaCl, pH 3.0). Replicate cultures were frozen at the indicated times after infection. The progeny virus was titrated in SK-OV-3-hN4 cells. For the cytotoxicity assay, triplicate cultures of hN4-high cells (MDA-MB-468, MCF-7, SK-BR-3, A-431, BxPC-3, CAPAN-1, SK-OV-3-hN4, LLC1-hN4, CT26-hN4, Renca-hN4) were seeded in 96 well plates 8 × 10^3^ cells/well and infected with R-421 or mock-infected. The input MOI was 0.05 PFU/cell according to the titer determined in the same cell line. AlamarBlue (Life Technologies) was added to the culture media (10 μL/well) at the indicated times after infection and incubated for 4 h at 37°C. Plates were read at 560 and 600 nm with GloMax Discover System (Promega) and data were processed according to the manufacturers’ instructions. For each time point, cell viability was expressed as the percentage of AlamarBlue signal reduction in infected versus uninfected cells, after subtraction of the background value (medium only).

### 2.6 Inhibition of R-421 infection by Abs to nectin4, to nectin1 or to HSV-1 gH/gL

For the infection blocking assay (Figure 4), replicate monolayers of SK-OV-3-hN4, human hN4-high cells (MDA-MB-468, MCF-7, SK-BR-3, A-431, BxPC-3, CAPAN-1), and murine LLC1-hN4, CT26-hN4, Renca-hN4 cells, seeded in 96-well plates, were preincubated with increasing amounts of PAb to nectin4 or of monoclonal antibody (MAb) R1.302 to nectin1 (Cocchi et al., 1998b) for 60 min at 37°C. R-421 (3 PFU/cell) was added to the medium containing the antibodies for additional 90 min. Alternatively, R-421 or R-LM5 virions (3 PFU/cell) were pre-incubated with increasing amounts of the HSV-1-neutralizing MAb 52S to gH/gL (Showalter et al., 1981) for 1 h at 37°C, and then allowed to adsorb to the cells for 90 min. After removal of the viral inoculum, cells were overlaid with medium containing the corresponding antibody (PAb to nectin4, MAb to nectin1, or MAb 52S). The extent of infection was quantified 18 h later by flow cytometry and expressed as the percentage of EGFP-positive cells relative to replicate cultures infected in the absence of the anti-Nectin4, anti-Nectin1 or 52S antibodies. The number of EGFP-positive cells in the cultures infected in absence of antibodies represented 100% of the infection.

### 2.7 *In vivo* experiments

C57BL/6 and BALB/c mice were obtained from Charles River Laboratories and bred in the facility of the Department of Veterinary Medical Sciences, University of Bologna. Both female and male mice were used randomly for the experiments, according to the request from the Ethical Committee. LLC1-hN4 cells were implanted subcutaneously in the left flank of 7-to-12 weeks old C57BL/6 mice in 100 μL of serum-free medium, 1 × 10^6^ cells/mouse. Similarly, CT26-hN4 or Renca-hN4 were implanted in the left flank of BALB/c mice, 1 × 10^6^ cells/mouse. Tumor volumes were scored 2–3 times weekly by measuring the largest and the smallest diameter by means of a caliper. Tumor volume was calculated using the formula: largest diameter x (smallest diameter)^2^ x 0.5. Mice were sacrificed after their tumors reached a volume of about 1,500 mm^3^, ulceration occurred, or animals exhibited distress or pain. When the tumor volumes averaged 70–100 mm^3^ (7–10 days post tumor engraftment for LLC1-hN4 cells, 10 days for CT26-hN4 cells, and 17 days for Renca-hN4 cells), mice received 2–3 intratumoral injections of R-421 (doses of 2 × 10^6^, 1 × 10^7^, 1.5 × 10^7^, 2.5 × 10^7^, 1 × 10^8^ PFUs–as detailed in figure legends–for each injection in 50 µL PBS) or vehicle (50 µL PBS), at 2–4 days intervals. The C57BL/6 mice that survived the primary tumor were subsequently engrafted with challenge tumors, specifically LLC1-hN4 (left flank) and LLC1-wt (right flank) cells, in 100 μL of serum-free medium, 1 × 10^6^ cells/mouse. The challenge tumors were not treated. Where indicated, mice received 3 intra peritoneal (i.p.) injections of anti-mouse PD-1 antibody (100 µg antibody in 100 µL PBS per mouse, clone RMP1-14, BioXcell), or vehicle (100 µL PBS) at 3–4 days intervals. For the immune cell depletion experiments, mice received 6 i.p. injections of either anti-mouse CD8a (clone YTS 169.4, BioXcell), CD4 (clone GK1.5, BioXcell), or NK (clone PK136, BioXcell) antibody 200 µg/mouse in 100 µL PBS, or vehicle (100 µL PBS) at 3–4 days intervals, starting 3 days before the first treatment with R-421. Five days after the last treatment with the immune depleting antibodies, blood samples were obtained from mice, immediately mixed with 2 mg/mL heparin (Merck), diluted with 5 mL cold FACS buffer, pelleted at 1,200 rpm for 7 min at 4°C, and resuspended in 1 mL of cold PBS. Cell suspensions were treated with 4 mL of ACK buffer (150 mM NH_4_Cl, 10 mM NaHCO_3_, 1 mM EDTA, cold) for 10 min in ice to lyse the red blood cells, then 8 mL of PBS were added, and samples were pelleted. Samples were resuspended in 50 µL staining solution: FACS buffer with CD45-PE-Cy7 (1.25 μg/mL; clone 30-F11, eBioscience), CD4-FITC (2.5 μg/mL; clone GK1.5, eBioscience), CD8a-PE (1 μg/mL; clone 53–6.7, eBioscience). Samples were incubated in ice for 30 min, then washed in FACS buffer and fluorescence was quantified by BD C6 Accuri. For the cyclophosphamide (CPA) treatment, mice received 2 i.p. injections of CPA (Merck), 2.5 mg/mouse in 250 µL PBS, or vehicle (250 µL PBS) at 7 days interval, starting 3 days before the first treatment with R-421.

### 2.8 Determination of serum antibodies to cancer cells

Mouse sera diluted 1:150 in FACS buffer were incubated with 1.5 × 10^5^ LLC1-wt or LLC1-hN4 cells, in 96 well plate for 1 h in ice. Cells were washed 3 times with ice-cold FACS buffer, incubated with anti-mouse APC (1:200, eBioscience) for 1 h in ice, and washed 3 times. Fluorescence was quantified by means of BD C6 Accuri.

## 3 Results

### 3.1 Engineering of human nectin4-retargeted R-421 recombinant and infection of human nectin4-positive cells

**FIGURE 1:**
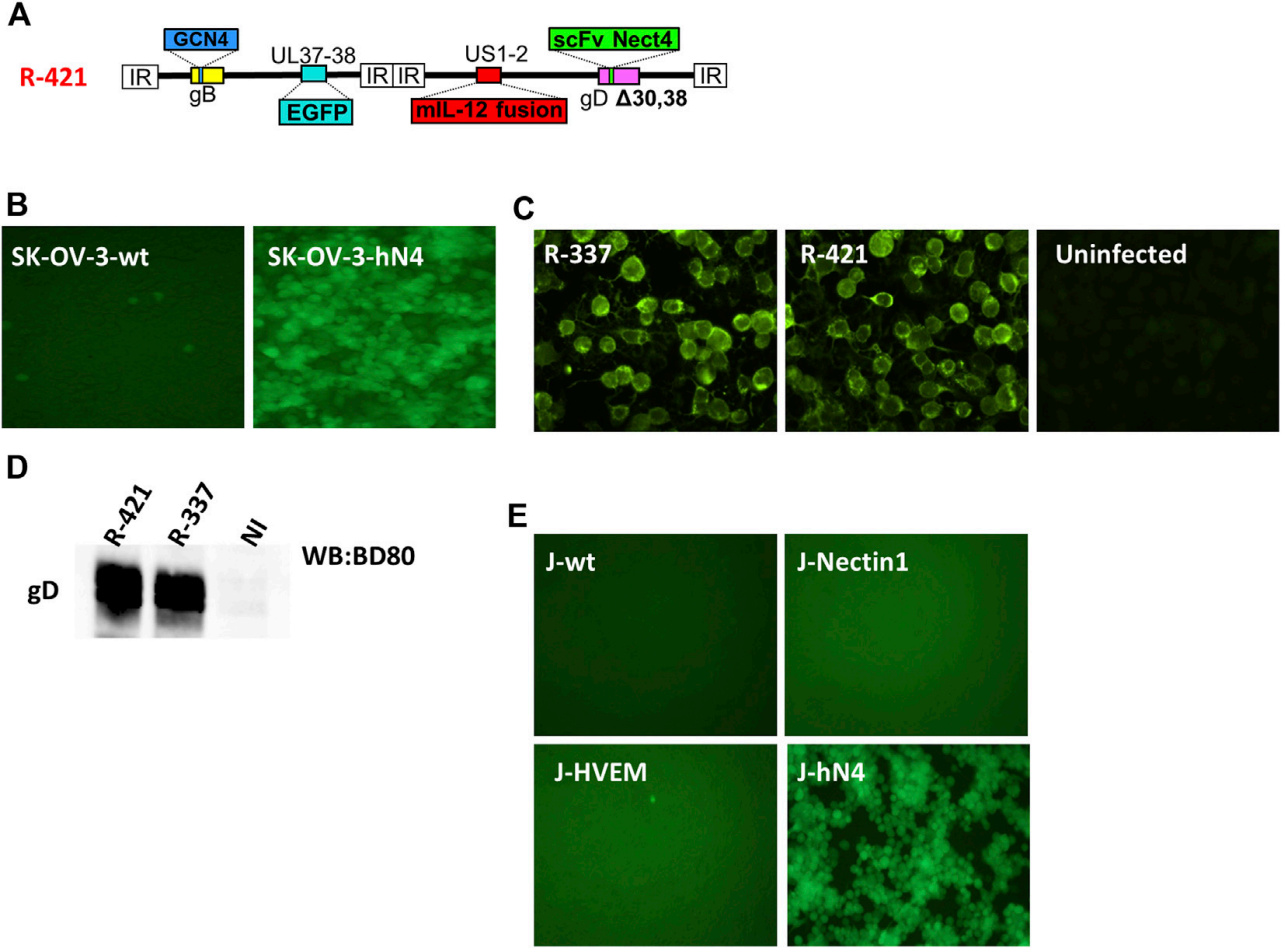
**(A)** Schematic representation of the genome of nectin4-retargeted R-421. Indicated are the genetic loci of gB, gD, the insertion of the GCN4 peptide in gB between aa 81 and 82, the insertion of mIL-12 in the US1 and US2 intergenic locus, the insertion of anti-nectin4 scFv for the retargeting to nectin4-positive cells, and the deletion of indicated amino acids in gD for the detargeting from HSV-1 natural receptors HVEM and nectin1. **(B)** Infection of SK-OV-3-hN4 cells, transgenic for human nectin4, and lack of infection of SK-OV-3-wt cells. Infection was detected by EGFP fluorescence. ×100 magnification. **(C)** Detection of gD protein in SK-OV-3-hN4 cells infected with R-337- or R-421, or uninfected by indirect immunofluorescence. Cells were infected with the indicated viruses (10 PFU/cell); 24 h later, cells were fixed with methanol (this treatment also quenched virus-encoded EGFP), reacted with anti-gD MAb BD80 and anti-mouse Alexa Fluor 488-conjugated secondary antibody. ×600 magnification. **(D)** Efficiency of gD incorporation in R-421 or R-337 virions. Purified virions were subjected to SDS-PAGE, transferred to nitrocellulose membranes, and visualized by Western blotting with the conformation-independent MAb BD80, directed against the C-terminus of gD ectodomain (264–274 aa). **(E)** R-421 infection of J cells expressing no HSV receptor (J-wt) or expressing the single receptor nectin1 (J-nectin1), HVEM (J-HVEM) or nectin4 (J-hN4).

R-421 is a derivative of the HER2-retargeted prototype ReHV named R-337 (Vannini et al., 2021b; Gianni et al., 2021). Briefly, the single chain antibody (scFv) to HER2 engineered in R-337 gD in place of amino acid (aa) 38 was replaced with a scFv to nectin4. The latter was synthesized based on the sequences of the variable light (VL) and heavy (VH) chains of the human α-nectin4 antibody (Lopez, 2018). For detargeting from the major herpes receptors nectin1 and HVEM, R-421 carries the single aa 30 and aa 38 deletions (Figure 1A). Like R-337, R-421 additionally carries the GCN4 peptide in gB to enable infection and growth in producer cells expressing the artificial GCN4 receptor (Gatta et al., 2015; Campadelli-Fiume et al., 2016b; Leoni et al., 2017; Petrovic et al., 2017; Leoni et al., 2018a; Petrovic et al., 2018), the EGFP (enhanced green fluorescent protein) engineered in the UL37-UL38 locus, the single peptide form of murine interleukin 12 (mIL12) in the US1-US2 intergenic locus (Figure 1A). To generate and grow R-421, we overexpressed human nectin4 (hN4) in the hN4-low SK-OV-3 human ovarian adenocarcinoma cell line that we routinely use for ReHV cultivation, thus generating SK-OV-3-hN4 cells. The hN4 median fluorescence intensities (MeFI) in SK-OV-3-hN4 and parental wt SK-OV-3 cells were about 293K and 4K, respectively (Table 1). Figures 1B shows that the SK-OV-3-hN4 cells were readily infected with R-421, whereas the wt SK-OV-3 parental cells exhibited only a few infected cells, as judged by EGFP expression. This provided the first line of evidence for retargeting to nectin4. To further validate SK-OV-3-hN4 as producer cells, we compared the level of gD expression in SK-OV-3-hN4 infected with R-421 or with R-337. Figures 1C, D show gD expression as detected by immunofluorescence or by Western blot and highlight similar extent of gD production in R-421- or R-337-infected cells.

**TABLE 1:** R-421 infection of malignant and non-malignant human and murine cell lines as a function of extent of human nectin4 expression.

	Cell line	Cancer from which cell line was derived	MeFI (K)^a^	Infection
Human	**SK-OV-3-hN4** ^b^	Ovary adenocarcinoma	293	Y
	**SK-BR-3**	Breast adenocarcinoma	31	Y
	**MCF-7**	Breast adenocarcinoma	30	Y
	**BxPC-3**	Pancreas adenocarcinoma	28	Y
	**MDA-MB-468**	Triple negative breast adenocarcinoma	24	Y
	**A-431**	Epidermoid carcinoma	22	Y
	**CAPAN-1**	Pancreas adenocarcinoma	11	Y
	U-2-OS	Osteosarcoma	5	N
	HeLa	Cervix adenocarcinoma	5	N
	SK-OV-3	Ovary adenocarcinoma	4	N
	U-251	Malignant glioblastoma multiforme	4	N
	K-562	Myelogenous leukemia	4	N
	MDA-MB-231	Triple negative breast adenocarcinoma	4	N
	PANC-1	Pancreas epithelioid carcinoma	4	N
	HPAC	Pancreas adenocarcinoma	3	N
	MRC-5	Normal fibroblasts	3	N
	HFF	Primary foreskin fibroblasts	1	N
Murine	LLC1	Lewis lung carcinoma	0.1	N
	Renca	Renal adenocarcinoma	0.1	N
	CT26	Colon carcinoma	0.1	N
	**LLC1-hN4** ^b^	Lewis lung carcinoma	426	Y
	**Renca-hN4** ^b^	Renal adenocarcinoma	169	Y
	**CT26-hN4** ^b^	Colon carcinoma	94	Y

^a^ Median fluorescence intensity detected as flow cytometry reactivity to α-nectin4 PAb.

^b^ = made transgenic for hN4.

To provide evidence of R-421 specific tropism for nectin4-positive cells, we carried out a gain-of-function experiment. A cell line transgenically expressing human nectin4 was generated in the background of the receptor-negative J cells resistant to HSV infection (Cocchi et al., 1998b), named J-hN4 cells. Moreover, J cells expressing the single nectin1 or HVEM receptor were employed to ascertain whether R-421 was detargeted from the natural receptors. Infection was monitored by means of EGFP. Figure 1E shows that indeed R-421 infected J-hN4 cells and failed to infect cells through the HSV natural receptors HVEM or nectin1, unlike the parental wt-HSV-1 strain (Cocchi et al., 1998b). R-421 also failed to infect the receptor-negative and HSV-resistant parental wt-J cells (Figure 1E) (Cocchi et al., 1998b).

**FIGURE 2:**
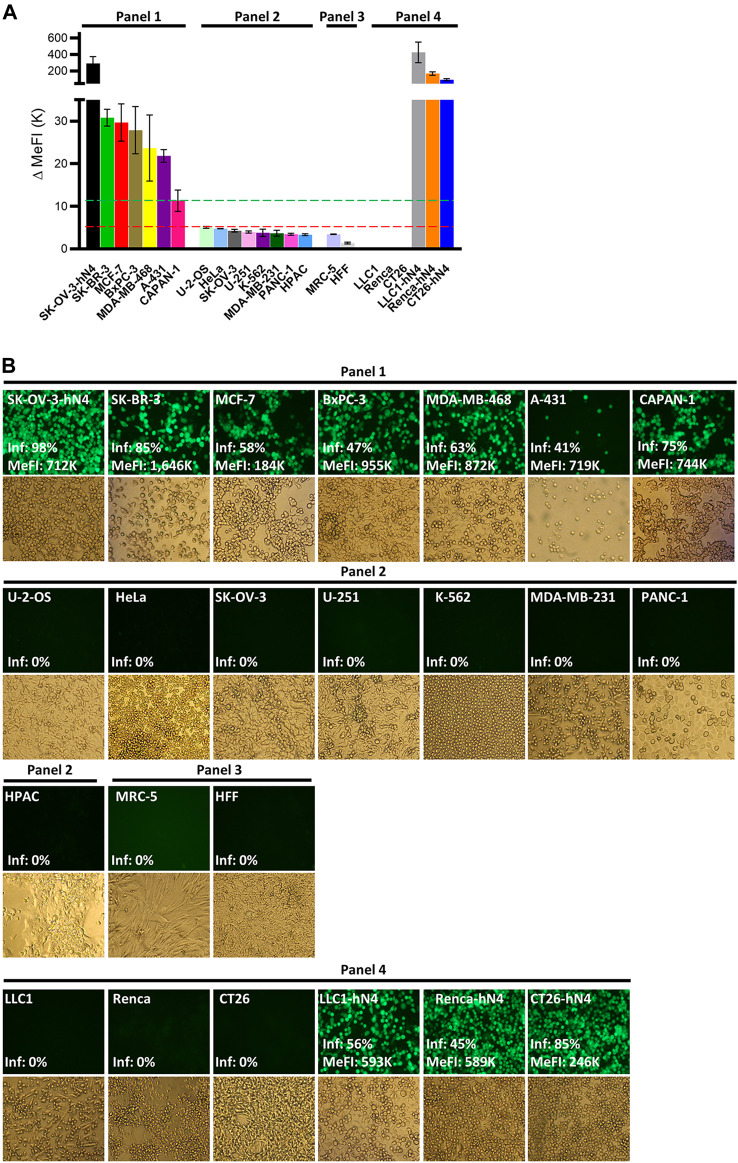
**(A)** Flow cytometry quantification of human nectin4 expression in the indicated cell lines. Cells were reacted with PAb to nectin4 or isotype control and then with the anti-rabbit Alexa Fluor 488-conjugated secondary antibody. Cells were grouped in Panel 1-4 according to the expression of hN4 and cell types. Panel 1 and Panel 2 include human malignant cell lines. Panel 3 includes human normal cell lines. Panel 4 includes murine tumor cell lines wt or transgenic for human nectin4. The hN4 MeFI threshold value for cell infection with R-421 is represented as a dashed green line. The MeFI value below which infection did not occur is reported as a dashed red line. Each bar represents the average of three determinations ± S.D. **(B)** R-421 infection of the indicated Panel 1-4 cells. For each cell line, the EGFP fluorescence and the brightfield images are shown. ×100 magnification. The EGFP fluorescence images report the percentage of infected (fluorescent) cells relative to total cells (Inf, %) and, where appropriate, the EGFP MeFI values of the infected (EGFP-positive) subpopulation, as determined in replicate cultures by flow cytometry. All fluorescence images were taken with a same exposure time (800 m-sec) and not modified; hence the EGFP signal in the images does not accurately reflect the relative intensities of infection across the various cell lines; these values are correctly expressed by flow cytometric determination as MeFIs.

To ascertain whether R-421 exhibited tropism for nectin4-positive human tumor cell lines, and to rule out off-target infection of nectin4-negative malignant and non-malignant cells, we preliminarily quantified nectin4 expression in three panels of cells by flow cytometry. Panel 1 (P1) included human tumor cells known to overexpress nectin4 (hN4-high), namely, BxPC3, SK-BR-3, MCF-7, A-431, MDA-MB-468, and CAPAN-1 (Uhlén et al., 2015). Panel 2 (P2) included human tumor cell lines known to express low levels of nectin4 (hN4-low), i.e., cells carrying no amplification or overexpression of nectin4, e.g., U-2-OS, HeLa, U-251, K-562, MDA-MB-231, PANC-1, and HPAC. Panel 3 (P3) included MRC-5 and HFF non-cancerous human cell lines and represented normal human tissues. Cancer cell lines in panels 1 and 2 were originally derived from triple negative and other types of breast cancers, pancreatic cancers, ovarian cancer, cervix cancer, epidermoid cancer, glioblastoma, osteosarcoma, and leukemia (Table 1). Figure 2A and Table 1 show the nectin4 MeFI in these cells, confirming the high expression level in the P1 hN4-high human malignant cells, and the moderate-to-low expression level in the malignant and non-malignant P2 and P3 hN4-low cells. Supplementary Figure S1 reports the percentage of nectin4-positive cells for each cell line, as determined by flow cytometry. Figure 2B and Table 1 show that all the human tumor cell lines whose nectin4 expression reached 11K or higher MeFI values (P1) were readily infected with R-421, as detected by EGFP fluorescence (see dashed green line in Figure 2A). Conversely, the human tumor cell lines whose nectin4 expression was at or below the 5K MeFI value (P2) were not infected with R-421 (see dashed red line in Figure 2A). These included MDA-MB-231 cells, which are classified as non-amplified nectin4-positive cells (Uhlén et al., 2015). We infer from this experiment that there is a threshold value of nectin4 expression that needs to be reached in order for cells to be infected with R-421 (Figure 2A) and cells whose expression is below the 5K MeFI value are not targeted by R-421. The exact threshold value for R-421 infection lies in between 11K and 5K MeFI values and remains to be determined more accurately. We note that CAPAN-1 cells, which express hN4 is at the threshold value (11K MeFI), are reported in Protein Atlas (Uhlén et al., 2015) as having a nTPM (normalized transcript per million) value of 10; U-2-OS cells, which express hN4 expression at 5K MeFI, are reported in Protein Atlas as having nTPM values 2.6 (Uhlén et al., 2015). Hence, the hN4 mRNA threshold value to grant infection with R-421 lies approximately in between 10 and 2.6 nTPM. Importantly, the non-malignant human HFF and MRC-5 cell lines (Figure 2 Panel 3; Table 1) failed to be infected with R-421. This finding rules out infection of non-malignant cells, likely reflecting a too low expression in normal cells. We note that among the Panel 1 hN4-high malignant cells, the extent of the EGFP expression varied (see MeFI values in Figure 2B panels for accurate determination). This likely reflects variations in co-receptors and factors that play a role in HSV entry downstream of gD receptors (Campadelli-Fiume et al., 2012) and variations in cell permissiveness, i.e., in post-entry restriction and defense factors, and in availability and half-life of cellular functions and factors required for viral transcription, translation and replication, etc. Altogether, we conclude from the experiments in Figures 1, 2 that.

• The expression of human nectin4 was a prerequisite for infection with R-421 (Figures 1B,E; Figures 2A,B).
• A predictive indicator of susceptibility to R-421 was the extent of nectin4 expression at or above the threshold value (green line). However, as expected, the extent and efficiency of infection was also affected by additional intrinsic features, e.g., variations in co-receptors and factors that play a role in HSV entry downstream of gD receptors and variations in permissiveness to HSV.
• Malignant cells were not intrinsically susceptible to R-421 unless they expressed nectin4 at or above the threshold value (green line).
• The non-malignant human fibroblast cells expressed nectin4 below the threshold value (red line) and were not infected with R-421.

The requirement for a certain degree of expression to enable cancer targeting by specific drugs is not at odds; thus, for example, patients are eligible for anti-HER2 antibody therapies if their HER2-positive breast cancer reach a certain predefined immunohistochemical score.

For subsequent experiments aimed at evaluating R-421 anticancer efficacy in immunocompetent mouse models, we expressed human nectin4 in murine tumor cells and originated transgenic CT26-hN4, Renca-hN4, and LLC1-hN4, syngeneic with BALB/c and C57BL/6 mice, respectively (Figure 2, Panel 4). Figures 2A,B show the high expression levels achieved in the hN4-transgenic murine cells, and document that they were infected with R-421. Their wt counterparts were not infected.

### 3.2 Efficiency of infection, cell-to-cell spread, replication and cytotoxicity of R-421 for hN4-high human malignant cells and for hN4-transgenic murine tumor cells

We selected a number of Panel 1 cells and the hN4-transgenic murine cancer cells to quantify how readily they were infected with and killed by R-421, the extent to which they enabled R-421 replication and cell-to-cell spread.

**FIGURE 3:**
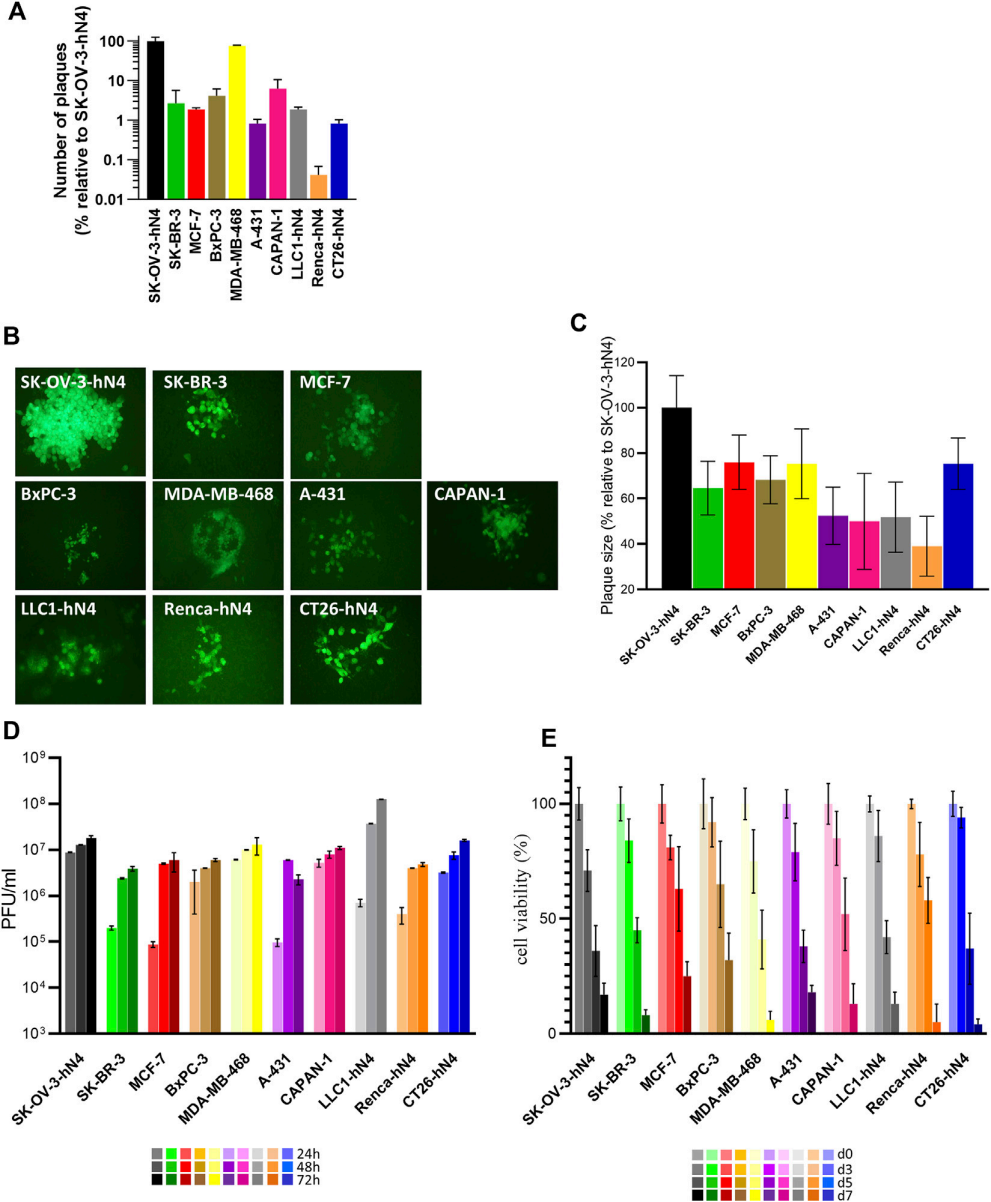
**(A)** Efficiency of R-421 infection. Replicate aliquots of R-421 were plated onto the indicated cell monolayers. The number of plaques formed in each cell line is expressed relative to the number of plaques formed in SK-OV-3-hN4 cells, taken as 100%. **(B)** Representative R-421 plaques in the indicated human or murine cell lines. ×100 magnification. **(C)** Average plaque size of R-421 in the indicated cell lines. For each of the indicated cell lines, ten pictures were taken, plaque areas were measured, expressed as percentage relative to plaque size in SK-OV-3-hN4 (indicated as 100%) and plotted ± SD. **(D)** Time course of R-421 replication in the indicated cells lines. Infected cells were harvested at 24, 48, and 72 h after infection at 0.1 PFU/cell, according to the titer determined in the same cell. Progeny virus was titrated in SK-OV-3-hN4 cells. Each column is the average of three samples ± standard deviation (SD). **(E)** Time course of cytotoxic effect of R-421 on the indicated cell lines. Cytotoxicity was determined by AlamarBlue as detailed (Petrovic et al., 2017) and expressed at each time point as the percentage of infected cells relative to uninfected cells. Each column is the average of three samples ± SD.

• The relative efficiency of R-421 infection in any given cell line (also referred to as plating efficiency in different cells) was quantified as the number of plaques formed by a fixed amount of R-421 relative to the number of plaques formed by the same amount of virus in SK-OV-3-hN4 cells; the latter value was taken as 100% (Figure 3A). The smaller the bar is, the lower the efficiency of infection is. The efficiency of infection varied greatly among the hN4-high human malignant cells (Figure 3A). The highest efficiency was seen in MDA-MB-468 cells, whose efficiency was similar to that of SK-OV-3-hN4 cells.
• The ability of the virus to spread from cell-to-cell was quantified as plaque size (Figures 3 B,C); it was highest in MDA-MB-468 and SK-OV-3-hN4 cells, and lowest in A-431 cells (Figures 3 B,C). Variations in the overall extent of EGFP expression across the plaques formed in different cell lines likely reflected in part the number of cells recruited to the plaque, and in part the factors that affected EGFP expression as noted in Figure 2B results.
• The extent and kinetics of R-421 replication was expressed as virus yield (PFU/mL) at 24, 48, 72 h after infection at MOI of 0.1 PFU/cell according to the titer in the same cell line (Figure 3D); in this assay, the various cell lines could not be significantly differentiated one from the other (Figure 3D).
• The ability of R-421 to kill the infected cells, as measured by Alamar Blue, did not greatly differ among the various human cancer cell lines (Figure 3E). Infection was carried out at a MOI of 0.05 PFU/cell, according to the titer in the same cell line.

The results document that R-421 was capable of high yield multiplication and cell-to-cell spread in the selected hN4-high malignant cells. The observed variations in the efficiency of infection and plaque size across hN4-high cell lines did not correlate with the extent of nectin4 expression, suggesting that hN4 levels over the threshold grant virus infection, and that additional cell-specific factors influence R-421 entry, replication, and cell-to-cell spread.

With respect to the murine tumor cells expressing hN4, LLC1-hN4 cells exhibited the highest ability to be infected and the highest yields of progeny virus (Figures 3 A,D). All hN4-expressing murine cells were killed by R-421 (Figures 3E).

### 3.3 Nectin4 serves as the critical portal of entry of R-421 and defines its tropism

**FIGURE 4:**
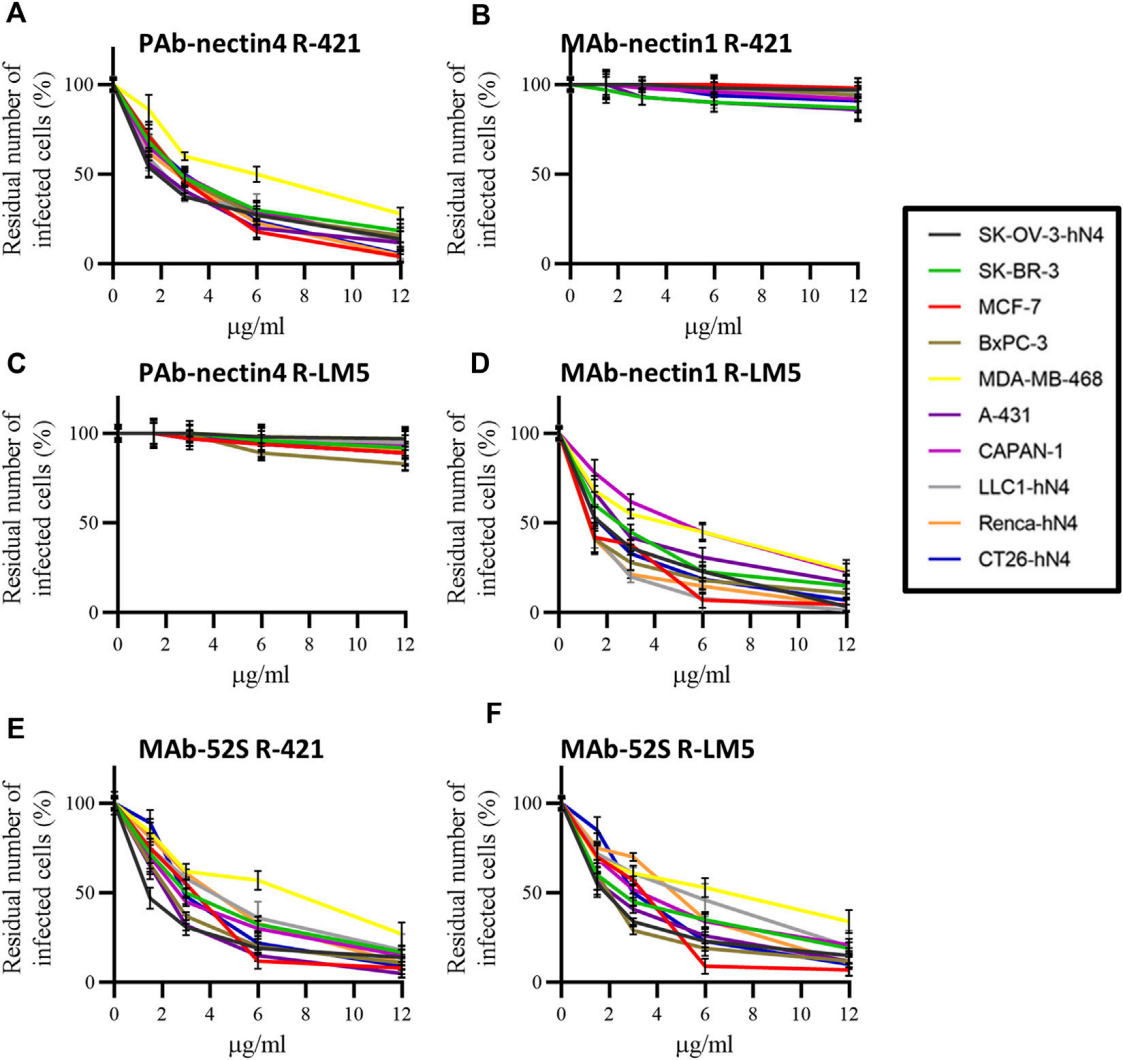
**(A–F)** Inhibition of R-421 or R-LM5 infection by increasing amounts of PAb to nectin4 **(A, C)**, MAb to nectin1 **(B, D)**, or MAb 52S to HSV gH **(E, F)**. The number of infected cells was quantified by flow cytometry. Each point represents the percentage of infected cells in the antibody-treated culture relative to the number of infected cells in the cultures not exposed to antibodies; the latter value was taken as 100%. Each point is the average of three samples ± SD.

Finally, we carried out an inhibition-of-function experiment to ascertain whether R-421 tropism for the nectin4-positive human tumor cells and for hN4-transgenic murine tumor cells was indeed dependent on the specific usage of nectin4 as the critical portal of entry. We also documented further the detargeting of R-421 from nectin1. Panel 1 hN4-high human cells were infected with R-421 (3 PFU/cell) in the presence of increasing amounts of PAb to nectin4 (PAb-N4) or MAb to nectin1 (MAb-N1). Infection was quantified 18 h later by flow cytometry. For each cell line, the 100% value represents the number of infected cells in the control culture, in which the infection was carried out in the absence of antibodies; each point in the curve represent the extent of inhibition of infection, i.e., the percentage of infected cells relative to the control. It can be seen that PAb-N4 reduced R-421 infection in a dose-dependent manner, whereas MAb-N1 failed to block infection (Figures 4 A,B). For comparison, we employed R-LM5, an essentially wt HSV that carries EGFP (Menotti et al., 2008). Its infection was blocked by MAb-N1 in all cells, as expected, and unaffected by PAb-N4 (Figures 4 C,D). The α-gH neutralizing MAb (52 S) (Showalter et al., 1981) blocked infection of either virus in all cells, as expected (Figures 4 E,F). The results document that nectin4 served as the *bona-fide* and the only functional receptor for R-421 in the hN4-high human malignant cells as well as in the hN4-transgenic murine tumor cells. Hence, R-421 exhibit specific tropism for nectin4-positive cells. The results provide additional evidence for R-421 detargeting from nectin1.

### 3.4 R-421 inhibits the growth of LLC1-hN4 tumors in immunocompetent mice

Of the three murine tumor cell lines expressing hN4, LLC1-hN4 ranked highest in terms of efficiency of infection and virus replication. CT26-hN4 exhibited reduced viral replication compared to LLC1-hN4 but were still efficiently infected. Renca-hN4 cells were reduced ten-fold or more in virus yield and infection efficiency compared to LLC1-hN4 cells.

**FIGURE 5:**
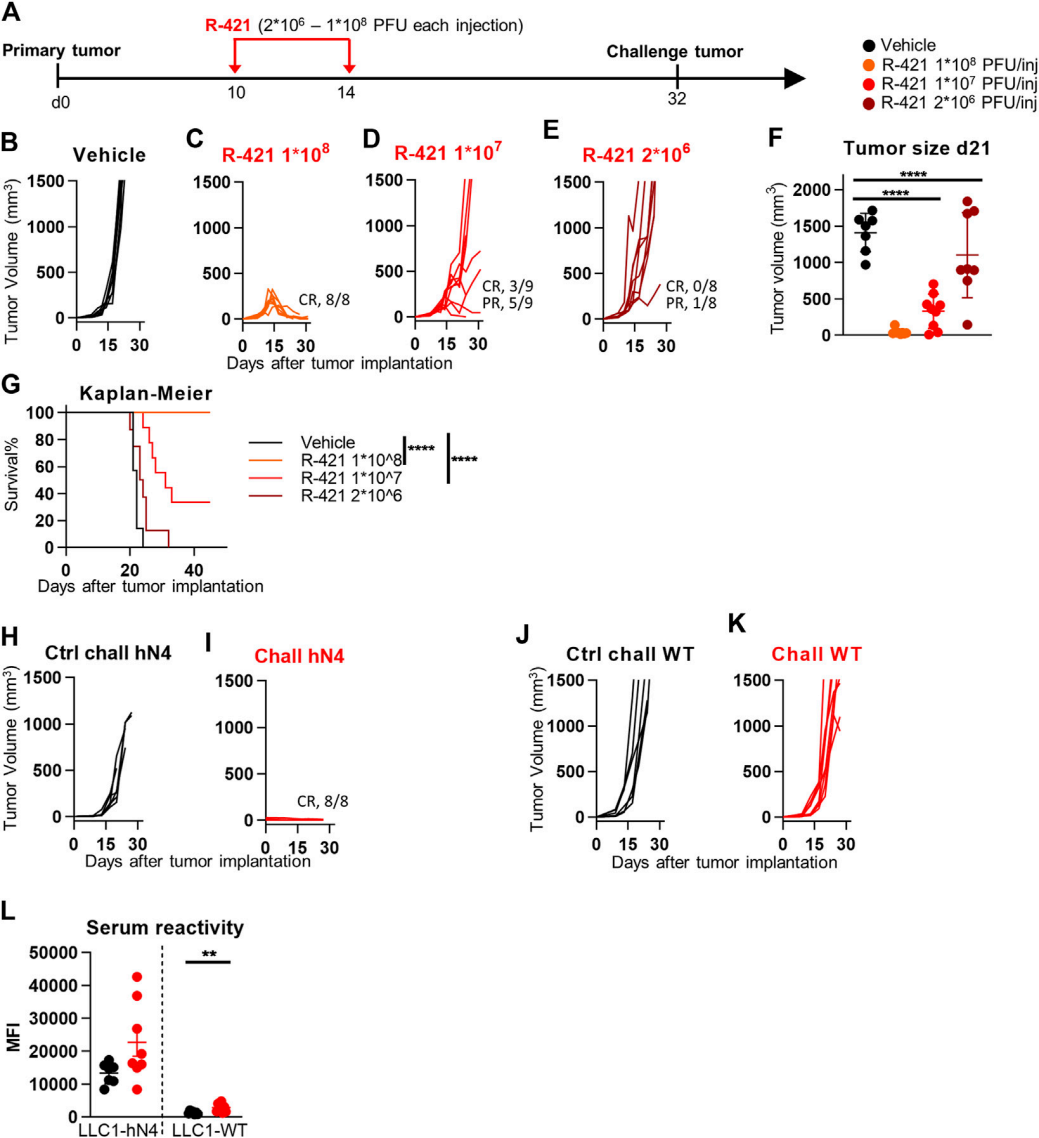
Efficacy of R-421 monotherapy on the growth of LLC1-hN4 tumors. **(A)** Schedule of treatments. 6-to-8 weeks old C57BL/6 mice were s.c. implanted in the left flank with 1 × 10^6^ LLC1-hN4 cells. 10 d later, when the tumor volumes averaged 70–100 mm^3^, mice received 2 intratumoral injections of R-421, ranging from 2 × 10^6^ to 1 × 10^8^ PFUs or vehicle, at 4 days interval. At d 32, mice treated with 1 × 10^8^ PFUs R-421 resulted tumor-free and received simultaneously two contralateral challenge tumors made of LLC1-hN4 and LLC1-wt cells (1 × 10^6^/mouse), respectively. **(B–E)** Tumor growth curves at the indicated dosages. The numbers reported in each panel indicate the numbers of mice that were completely cured from tumors (complete response, CR), or that showed a delay/reduction in tumor growth (partial response, PR). Mice were scored PR when the tumor volume was <50% smaller than the mean size of the tumors in the vehicle group, in at least 2 consecutive measurements. **(F)** Volumes of the primary tumors at d 21 after implantation. **(G)** Kaplan-Meier survival curves of the four groups of mice. **(H–K)** Kinetics of growth of challenge tumors in naïve mice **(H, J)**, or in the R-421 survivors’ arm **(I, K)**. Mice received simultaneously LLC1-hN4 **(H, I)** and LLC1-wt cells **(J, K)** in the left and right flanks, respectively. Absence of tumor growth is indicated as CR. **(L)** Antibodies to LLC1-hN4 or LLC1-wt cells in sera harvested at sacrifice. Reactivity was measured in CELISA test. **(F, L)** Each circle corresponds to an individual mouse; the horizontal line indicates the mean value, and vertical bars ± SD. **(F, G, L)** Statistical significance was calculated by t-test **(L)**, ANOVA test with Tukey’s correction **(F)**, or Log-rank (Mantel-Cox) test with Bonferroni’s correction **(G)** and expressed as ** = *p*-value < 0.01; **** = *p*-value < 0.0001. Color code: mice treated with vehicle or R-337 are indicated in black or red, respectively.

We performed an escalating dose-efficacy experiment in C57BL/6 mice implanted subcutaneously with LLC1-hN4 cells. R-421 was administered intratumorally (i.t.) in two doses at day 10—when tumors reached an average size of 70–100 mm^3^—and at day 14. At each dose, the amount of R-421 was 2 × 10^6^, 1 × 10^7^, or 1 × 10^8^ PFUs (schedule depicted in Figure 5A). Figure 5B–E shows the tumor growth curves and reports the number of mice exhibiting CR (complete response) or PR (partial response). The highest dose conferred CR in 100% of the mice (Figure 5C). The 1 × 10^7^ dose (Figure 5D) resulted in CR and PR in 3/9 and 5/9 mice, respectively. The lowest dose was not effective (Figure 5E). The reduction in tumor size at day 21 was highly significant for the two higher doses (Figure 5F). The Kaplan-Meier survival curve shows highly significant differences between each of the two higher doses and vehicle (Figure 5G). The mice that received the highest amount of R-421 (Figure 5 C–CR in 100%) were subsequently implanted with two distant challenge tumors made of LLC1-hN4 and LLC1-wt cells, respectively. Figures 5H–K shows that none of the mice developed the LLC1-hN4 challenge tumor (Figure 5I); all developed the LLC1-wt tumor (Figure 5K). The antibody response measured at sacrifice revealed immunoreactivity to LLC1-hN4 cells was higher than to LLC1-wt cells (Figure 5L) and increased in the R-421-treated animals. Inasmuch as none of the challenge tumors were treated, protection from tumor growth was immune mediated and resulted from the systemic response elicited by the *in situ* vaccination of primary tumors. Altogether, the results indicate that R-421 exerted anticancer efficacy in a dose-dependent manner and elicited an *in situ* vaccination to LLC1-hN4 cells that prevented the growth of distant LLC1-hN4 challenge tumors.

### 3.5 R-421 dramatically augments immune checkpoint inhibition in combination therapy

**FIGURE 6:**
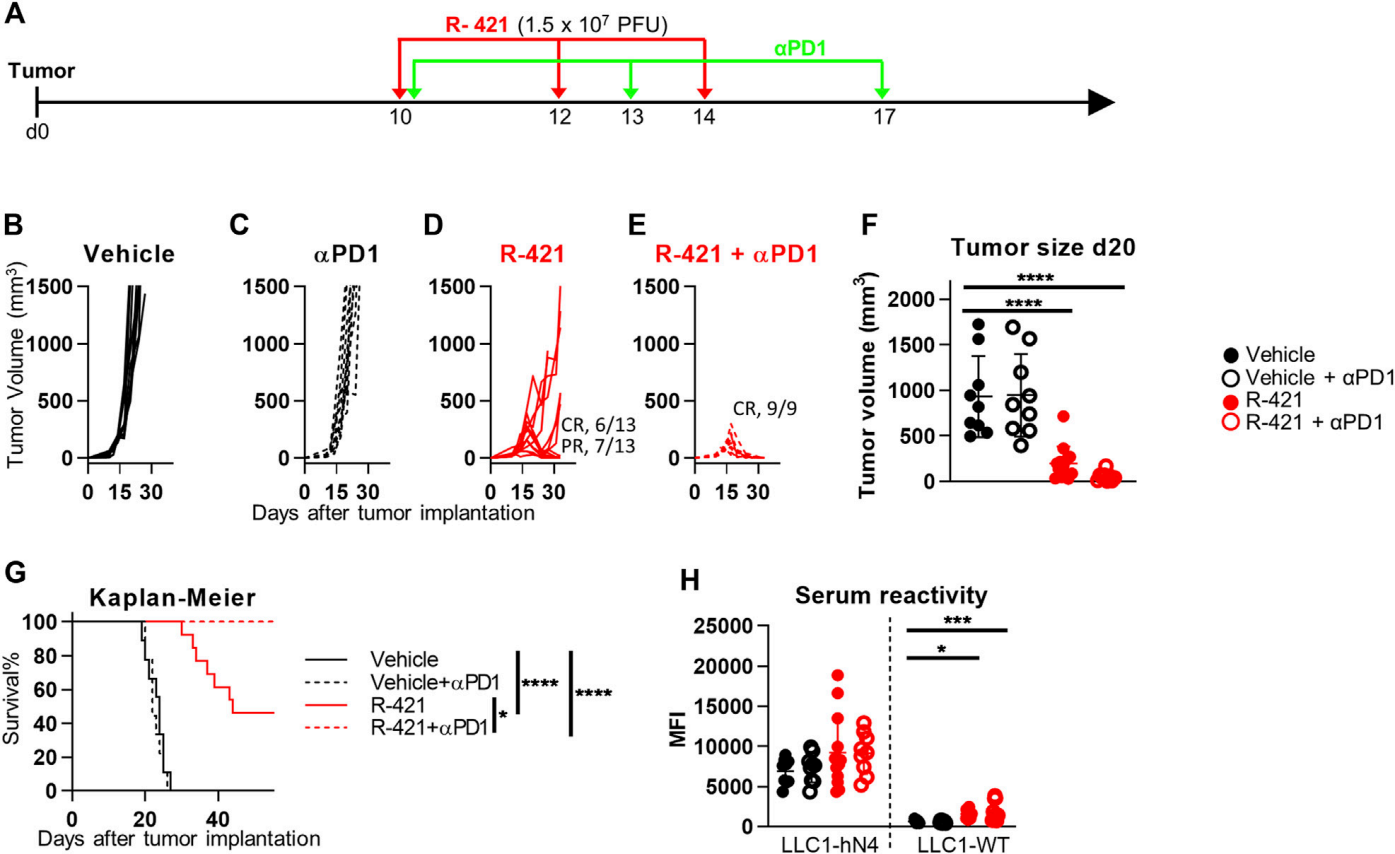
Therapeutic effects of R-421 in combination with anti-PD-1 antibody (αPD1) on the growth of LLC1-hN4 tumors. **(A)** Schedule of the treatments. Mice were implanted with LLC1-hN4 cells. At d 10 after implantation, when tumors reached the average volume of 70–100 mm^3^, mice received 3 i.t. injections of R-421 (1.5 × 10^7^ PFUs) or vehicle at 2 days intervals and 3 i.p. injections of MAb to PD-1 (αPD1) at 3–4 d intervals. **(B–E)** Tumor growth curves at indicated dosages. **(F)** Volumes of the primary tumors at d 20 after implantation. **(G)** Kaplan-Meier survival curves of the four indicated groups of mice. **(H)** Long-term B cell immunity to LLC1 tumors measured as serum antibodies to LLC1-hN4 and LLC1-wt cells. **(F–H)** Statistical significance was calculated by means of ANOVA test with Tukey’s correction **(F, H)** or Log-rank (Mantel-Cox) test with Bonferroni’s correction **(G)** and expressed as * = *p*-value < 0.05; *** = *p*-value < 0.001; **** = *p*-value < 0.0001. Color codes: mice treated with vehicle (black) or R-421 (red). Full circles and continuous lines, no αPD1 antibody. Open circles and dotted lines, employment of αPD1 antibody.

Next, we investigated whether treatment with R-421 confers efficacy to the immunotherapy with antibodies to PD-1 (αPD1). Mice implanted with LLC1-hN4 tumors were treated with suboptimal amounts of R-421 administered i.t. in three doses, as monotherapy or in combination with αPD1 (schedule depicted in Figure 6A). Figures 6B–E show that while the R-421 monotherapy at this dosage resulted in CR in 46% of the mice and in PR in the remaining mice (Figure 6D), the combination therapy resulted in CR in 100% of the mice (panel E). The αPD1 monotherapy did not inhibit tumor growth (Figure 6C). Tumor size at d 20 (Figure 6F) and the Kaplan-Meier survival curve (Figure 6G) recapitulated these effects and further showed the significance of the difference between R-421 monotherapy and ICI combination therapy. Analysis of serum antibodies showed that both R-421 monotherapy and combination therapy elicited B cell response towards LLC1-hN4 cells and to a lesser extent to LLC1-wt cells (Figure 6H). Overall, the results extend to nectin4 the notion that ReHV treatment confers αPD1 sensitivity to intrinsically resistant tumors (Lichty et al., 2014; Kaufman et al., 2015; De Lucia et al., 2020; Vannini et al., 2021b; Gianni et al., 2021; Sitta et al., 2022) and indicate that in the combination therapy a lower amount of ReHV was required to achieve CR response in 100% of mice.

### 3.6 R-421 moderately reduces the growth of CT26-hN4 and less so of Renca-hN4 tumors

**FIGURE 7:**
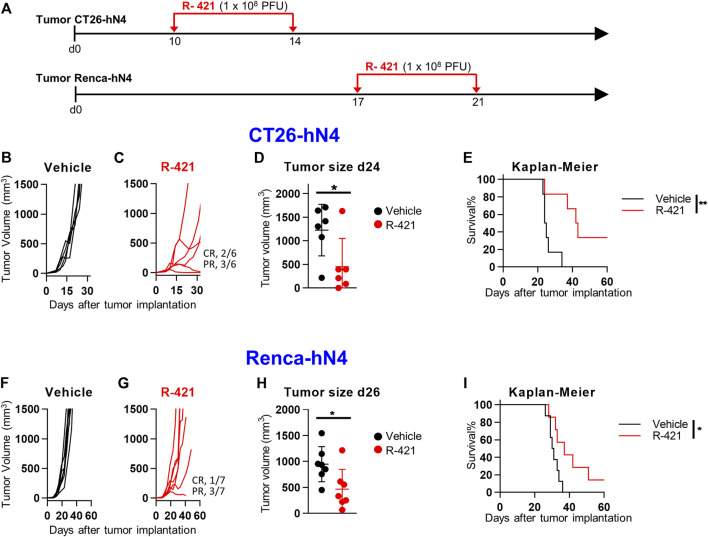
Efficacy of R-421 monotherapy on the growth of CT26-hN4 and Renca-hN4 tumors. **(A)** Schedule of monotherapy treatment. Mice were s.c. implanted in the left flank with 1 × 10^6−ΔΔCT^26-hN4 or Renca-hN4 cells. 10 d (CT26-hN4) or 17 d later (Renca-hN4), respectively, when the tumor volumes averaged 70–100 mm^3^, mice received 2 i.t. injections of R-421 (1 × 10^8^ PFUs) or vehicle at 4 days interval. **(B–E)** R-421 efficacy in CT26-hN4 tumors, with tumor growth curves **(B, C)**, volumes of the primary tumors at d 24 after implantation **(D)**, and Kaplan-Meier survival curves **(E)**. **(F–I)** R-421 efficacy on the growth of Renca-hN4 tumors, with tumor growth curves **(F, G)**, volumes of the primary tumors at d 26 after implantation **(H)**, and Kaplan-Meier survival curves **(I)**. **(D,E,H,I)** Statistical significance was calculated by means of t-test **(D, H)** or Log-rank (Mantel-Cox) test **(E, I)**, and expressed as * = *p*-value < 0.05; ** = *p*-value < 0.01.

We asked how effective R-421 was against CT26-hN4 and Renca-hN4 tumor cells, which enable the virus infection and replication at lower efficiency than LLC1-hN4 cells. CT26-hN4 or Renca-hN4 tumor cells were implanted s.c. into BALB/c mice; 10 and 14 d later (17 and 21 d for Renca-hN4), R-421 was administered i.t. to the grown tumors in two doses of 1 × 10^8^ PFU each (schedule depicted in Figure 7A). Figures 7B,C show the CT26-hN4 tumor growth curves; the treatment resulted in 2/6 and 3/6 mice exhibiting CR or PR, respectively. CT26-hN4 tumor sizes at d 24 reported in Figure 7D and the Kaplan-Meier survival curves showed a certain protection against CT26-hN4 tumors (Figure 7E). The effect was even lower for Renca-hN4 tumors, with 1/7 and 3/7 mice exhibiting CR and PR, respectively (Figures 7 F,G), a lesser but still significant reduction of tumor size at d 26 (Figure 7H). The Kaplan-Meier survival curves showed a protection against Renca-hN4 tumors (Figure 7I) lower than those observed for CT26-hN4 and LLC1-hN4. The results are in agreement with the *in vitro* characteristics of the two tumor cells reported in Figure 3, that indicated that Renca-hN4 cells enabled R-421 replication at tenfold or lower yields and required ten-fold higher amounts of R-421 than LLC1-hN4 or CT26-hN4 cells. Overall, the lower *in vivo* protection mirrored the lower capacity of CT26-hN4 and, especially, of Renca-hN4 cells to enable R-421 infection and replication.

### 3.7 R-421 efficacy against LLC1-hN4 tumors is increased upon cyclophosphamide (CPA) treatment

**FIGURE 8:**
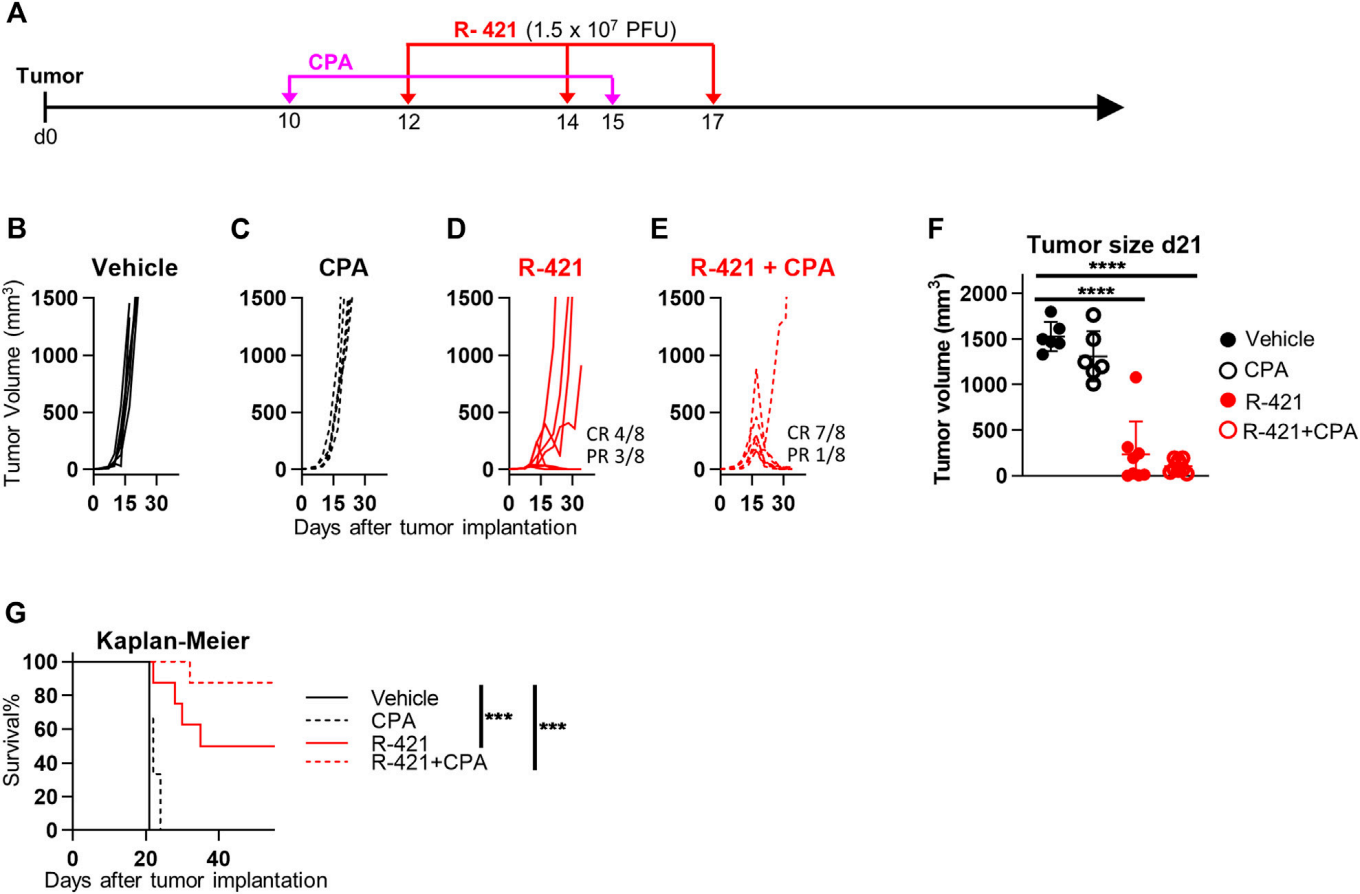
Therapeutic effects of R-421 in combination with cyclophosphamide (CPA) on the growth of LLC1-hN4 tumors. **(A)** Schedule of the treatments. Mice were implanted with LLC1-hN4 cells. At d 10 after implantation, when tumors reached the average volume of 20–30 mm^3^, mice received 2 i.p. injections of CPA at 9 days interval, and when tumors reached 70–100 mm^3^ (d 12) mice received 3 i.t. injections of R-421 (1.5 × 10^7^ PFUs) or vehicle at 2–3 days intervals. **(B–E)** Tumor growth curves at indicated mono- or combination therapies. **(F)** Volumes of the primary tumors at d 21 after implantation. **(G)** Kaplan-Meier survival curves of the four indicated groups of mice. **(F–G)** Statistical significance was calculated by means of the ANOVA test with Tukey’s correction **(F)** or Log-rank (Mantel-Cox) test with Bonferroni’s correction **(G)** and expressed as *** = *p*-value < 0.001; **** = *p*-value < 0.0001. Color codes: mice treated with vehicle (black) or R-421 (red). Full circles and continuous lines, no CPA. Open circles and dotted lines, employment of CPA.

Numerous efforts are ongoing in preclinical and clinical settings to spur the efficacy of oncolytic or onco-immunotherapeutic viruses by combining them with drugs or treatments that boost the immune response or favor viral replication. One such drug is CPA, a DNA alkylating agent, that acts as an immunomodulator and as an anticancer chemotherapeutic. Studies by Chiocca and collaborators have elegantly shown that CPA increases oncolytic herpesvirus replication within the tumor bed and their anticancer efficacy (Kasai et al., 2013; Chiocca et al., 2020); the combination is currently being evaluated in a clinical trial. Here we tested whether R-421 efficacy against LLC1-hN4 tumors was increased by co-treatment with CPA. Mice bearing LLC1-hN4 tumors were treated with suboptimal amounts of R-421 alone or in combination with i.p. CPA (Figure 8A). Figures 8B–E show that CPA monotherapy had no significant effect on tumor growth at the selected regimen. R-421 monotherapy exerted CR in 50% of the mice, while the combination exerted CR in 88% of the mice; the results were confirmed in tumor volume determinations at d 21 (Figure 8F) and in the Kaplan-Meier survival curve (Figure 8G). Thus, a combination regimen with CPA might be employed to boost R-421 efficacy and to decrease the amount of virus to be employed *in vivo*.

### 3.8 The anti-tumor efficacy of R-421/αPD1 combination is T cell-mediated and dependent on CD8-positive lymphocytes

**FIGURE 9:**
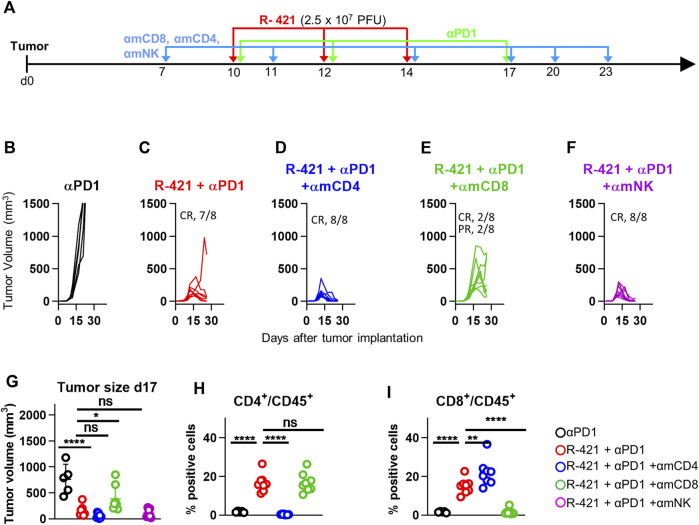
Therapeutic effects of the R-421 and αPD1 combination on the growth of LLC1-hN4 tumors in mice depleted of specific sub-populations of immune cells (CD4, CD8, NK). **(A)** Schedule of the treatments. Mice were implanted with LLC1-hN4 cells. At d 7 after implantation, when tumors reached the average volume of 20–30 mm^3^, mice received 6 i.p. injections either of αCD4, αCD8, or αNK at intervals of 3–4 days to deplete the corresponding immune cells. At d 10 after implantation, when tumors reached the average volume of 70–100 mm^3^, mice received 3 i.t. injections of R-421 (2.5 × 10^7^ PFUs) or vehicle at 2 days intervals and 3 i.p. injections of αPD1 at 3–4 d intervals. **(B–F)** Tumor growth curves at the indicated treatments and immune cells depletions. **(G)** Volumes of the primary tumors at d 17 after implantation. **(H, I)** Validation of CD4 and CD8 depletions. Immune cells were isolated from blood samples, then stained to detect CD45+, CD4+CD45+ and CD8+CD45+ subpopulations. Percentage of CD4-positive **(H)** or CD8-positive cells **(I)** in the CD45-positive cells. **(G–I)** Statistical significance was calculated by means of ANOVA test with Tukey’s correction, employing the R-421 + αPD1 no immune depleted group (red) as reference, and expressed as * = *p*-value < 0.05; ** = *p*-value < 0.01; **** = *p*-value < 0.0001. Color codes: mice treated with αPD1 antibody (black), R-421 + αPD1 (red), R-421 + αPD1 with the depletion of CD4 (blue), CD8 (green), or NK cells (purple). As detailed in Supplementary Figure S2, cells were gated for cell size on an SSC vs. FSC plot in the range of 1,000,000-5,000,000 (FSC) and 0-600,000 (SSC). Cells reacted with isotype controls or single staining with MAb for CD45 were used to select the plot areas for CD45+, CD4+CD45+, and CD8+CD45+ populations.

Numerous lines of evidence have demonstrated that the anticancer efficacy of oncolytic viruses, including retargeted oncolytic herpesviruses, is immune mediated and that their treatment induces anticancer vaccination (Hughes et al., 2014; Lichty et al., 2014; Leoni et al., 2018b; De Lucia et al., 2020). Here we investigated the mechanism of anti-tumor responses, in particular the contribution of the major immune cell subpopulations. Mice bearing LLC1-hN4 tumors and treated with R-421 and αPD1 were selectively depleted of the CD4-positive, CD8-positive, or NK (natural killer) cell subpopulations by means of appropriate antibodies (Schedule in Figure 9A). Figures 9B–F show that virus/αPD1-mediated tumor growth inhibition was unaffected by the depletion of the CD4-positive subpopulation, (panel D) and, conversely, was partially released upon CD8-positive cells depletion (Figure 9E). Depletion of NK cells did not apparently restore tumor growth (Figure 9F). This trend was confirmed by the evaluation of tumor volumes at d 17 (Figure 9G). The effective depletion of CD4-and CD8-positive subpopulations was confirmed (Figures 9 H,I). While the depletion of the CD4-population may have affected both the antitumoral T-helpers and the pro-tumoral T-regulatory lymphocytes and the net effect remains to be investigated in more detail, the results underscore a clear contribution of the effector CD8-positive subpopulation to tumor clearance in this system, in agreement with previous results (De Lucia et al., 2020). This series of data compellingly shows that the T response is critical to the R-421-mediated anti-cancer therapy.

## 4 Discussion

The development of nectin4–specific drugs is in its infancy. So far, Enfortumab Vedotin is the first-in-class and the only approved therapeutic (Chang et al., 2021). Only five clinical trials are currently investigating nectin4 as therapeutics’ target. To generate an innovative anti-nectin4 agent, we engineered R-421, a novel oncolytic herpesvirus with specific tropism for nectin4 and detargeted from the major natural HSV receptors nectin1 and HVEM. Virus retargeting was documented in gain-of-function and inhibition-of-function experiments, as well as by the virus ability to infect solely nectin4-positive cells. In particular, R-421 acquired the ability to infect receptor-negative cells upon transfection of human nectin4, and infection of human cells was inhibited by anti-nectin4 antibodies, indicating that the only functional pathway of entry was the one dependent on nectin4. R-421 detargeting was confirmed by the inability of the virus to infect cells expressing nectin1 or HVEM receptors, or normal human fibroblast lines (HFF and MRC-5), representative of normal human tissues. R-421 was armed with a single peptide form of murine IL-12 to boost its immunotherapeutic effects. *In vitro*, R-421 specifically targeted and killed a number of nectin4-positive human malignant cells, representative of clinically relevant nectin4-positive indications, in particular triple negative breast cancers, pancreatic ductal carcinomas, breast and epidermoid carcinomas.

From the point of view of translation to the clinic, worth of note was the finding that not all malignant cells positive for nectin4 became infected with R-421. Thus, cells known to be weakly positive but to not carry nectin4 gene amplification (moderate-to-low expression), exemplified by MDA-MB-231, HPAC, and PANC-1 cells, were not infected. Essentially, nectin4 was a requirement for R-421 infection to occur, although its mere presence was not sufficient for infection. A predictive indicator for susceptibility to infection was the extent of nectin4 expression above the threshold value in otherwise permissive cells. The threshold value for susceptibility to infection appears to be a critical safety feature that may favor the translation of nectin4-retargeted oncolytic herpesviruses to the clinic, as it predicts that, in humans, the non-malignant tissues that express nectin4 at low level—i.e., do not harbor nectin4 gene amplification—are spared by R-421. Essentially, off-tumor in-target infections of normal tissues should not take place. Given its ability to infect solely nectin4 overexpressing cells, R-421 has the potential to not cause the adverse effects that occasionally mar Enfortumab Vedotin (Wu and Adamson, 2019; Enescu et al., 2022). The requirement for a certain degree of expression in order for a target to be druggable is not at odds and is part of clinical practice. For example, in order for patients to be eligible for anti-HER2 antibody therapy (e.g., trastuzumab, or pertuzumab), the HER2-positive breast cancers must have a predefined immunohistochemical score.

We set up immunocompetent murine models of human nectin4-positive tumors by transgenically expressing hN4 in LLC1 cells, an immunologically desert type of tumor syngeneic with C57BL/6, and in the immunogenic CT26 and Renca tumors, syngeneic with BALB/c mice. LLC1-hN4 cells supported R-421 infection, replication and spread to higher yields than the CT26-hN4 cells, which, in turn, were better infected than Renca-hN4 cells. The latter were particularly limited in spreading from infected cells to the adjacent ones. R-421 was cytotoxic *in vitro* to all these cells. *In vivo*, R-421 monotherapy inhibited LLC1-hN4 tumors in a dose dependent manner; at the highest dose, R-421 exerted a complete response in 100% of mice. The surviving mice were then challenged with distant LLC1-hN4 tumors and were fully protected from them. Oncolytic viruses are important coadjutors of immune checkpoint inhibitors (Topalian et al., 2011; Lichty et al., 2014; Bommareddy et al., 2018; Senior, 2019; Vannini et al., 2021a); thus, tumors that do not respond or respond moderately to ICIs become sensitive to ICIs, as a result of the dramatic modifications that OVs induce to tumor microenvironment. LLC1-hN4 tumors, which were highly resistant to antibodies to PD-1, became sensitive when treated with the R-421 and αPD1 combination therapy. Treatment of CT26-hN4, and even more of Renca-hN4, was much less effective than treatment of LLC1-hN4 tumors, most likely reflecting the high replication and cell-to-cell spread of R-421 in the latter cells. It is worth noting that although murine cells and mice sustain replication of HSV, a human virus, some murine cell lines and some mouse strains are highly resistant to HSV (Lopez, 1975). Indeed, it has been routinely observed that effective doses of oncolytic herpesviruses are much lower in humans than in mice (Andtbacka et al., 2015; Moesta et al., 2017). This is also true for the retargeted oncolytic herpes viruses, which were much more effective towards human than murine tumors (Menotti et al., 2009; Leoni et al., 2018b), despite the fact that the efficacy towards the human tumors was tested in nude mice, i.e., in the absence of immunotherapeutic effects. These findings predict that anticancer effects of nectin4-ReHVs should be much higher (and the effective doses much lower) towards N4-high human cancers than those reported here in murine models.

The finding of nectin4 as a novel cancer antigen along with the clinical relevance of the nectin4-positive indications is spurring the search for nectin4-targeting treatments, including OVs. The pathogenetic ubiquitous measles virus (MeVs) strains enter cells through SLAM (signaling lymphocyte-activation molecule) and human nectin4, hence have a natural tropism for hN4. In contrast, the vaccine strains preferentially employed to generate oncolytic MeVs infect cells through CD46 (Dhiman et al., 2004; Muhlebach et al., 2011). A single aa substitution in MeV wt hemagglutinin attenuated its capacity to use SLAM receptor and left the ability to use nectin4. The SLAM-blind MeV recombinant was cytotoxic *in vitro* for nectin4-positive cells derived from pancreatic, colorectal and triple negative cancer cells. *In vivo*, it inhibited tumor growth in xenograft models in nude mice (Amagai et al., 2016; Awano et al., 2016; Fujiyuki et al., 2020). A notable difference between the SLAM-blind MeV system and current nectin4-retargeted herpesvirus appears to be that the MeV recombinant infected all hN4-positive cancer cells regardless of their extent of nectin4 expression; a modest infection was reported also with some cells whose nectin4 expression was negative or below the detection limit. The behavior toward non-malignant cells, the stability of the mutation-based SLAM-blindness, and the long-lasting vaccination did not appear to have been investigated to date, nor was a threshold value for nectin4 expression in malignant cells reported.

This study provides proof-of-principle specificity and efficacy data justifying human nectin4-retargeted onco-immunotherapeutic herpesvirus as an innovative approach against a number of difficult-to-drug clinical indications.

## Data Availability

The original contributions presented in the study are included in the article/Supplementary Material, further inquiries can be directed to the corresponding authors.
