# Corrigendum: Innovative retargeted oncolytic herpesvirus against nectin4-positive cancers

**DOI:** 10.3389/fmolb.2024.1444446

**Published:** 2024-06-25

**Authors:** Andrea Vannini, Federico Parenti, Cristina Forghieri, Catia Barboni, Anna Zaghini, Gabriella Campadelli-Fiume, Tatiana Gianni

**Affiliations:** ^1^ Department of Medical and Surgical Sciences (DIMEC), University of Bologna, Bologna, Italy; ^2^ Department of Pharmacy and Biotechnology, University of Bologna, Bologna, Italy; ^3^ Department of Veterinary Medical Sciences, University of Bologna, Bologna, Italy

**Keywords:** oncolytic virus, oncolytic herpes simplex virus, retargeting, nectin4, anti-cancer vaccine, immunotherapy, pancreas carcinoma, triple negative breast cancer

In the published article, the **Reference** for Lopez and Olive, 2020 was incorrectly written as “Lopez, M., and Olive, D. (2020). Antibodies having specificity to nectin-4 and uses thereof. Google Patents”. It should be “Lopez, M. (2018) Antibodies having specificity to nectin-4 and uses thereof. Google Patents.”

The authors apologize for this error and state that this does not change the scientific conclusions of the article in any way. The original article has been updated.

