# Supplementary material for: Innovative retargeted oncolytic herpesvirus against nectin4-positive cancers
Source: Front Mol Biosci. 2023 May 11;10:1149973. doi: 10.3389/fmolb.2023.1149973 (PMC10213976; doi:10.3389/fmolb.2023.1149973)
Supplement: Supplementary file 1 [file DataSheet1.DOCX]

Innovative retargeted oncolytic herpesvirus against nectin4-positive cancers

Andrea Vannini, Federico Parenti, Cristina Forghieri, Catia Barboni, Anna Zaghini, Gabriella Campadelli-Fiume*† and Tatiana Gianni1*†

*** Correspondence:** gabriella.campadelli@unibo.it (G.C.F.); tatiana.gianni3@unibo.it (T.G.)


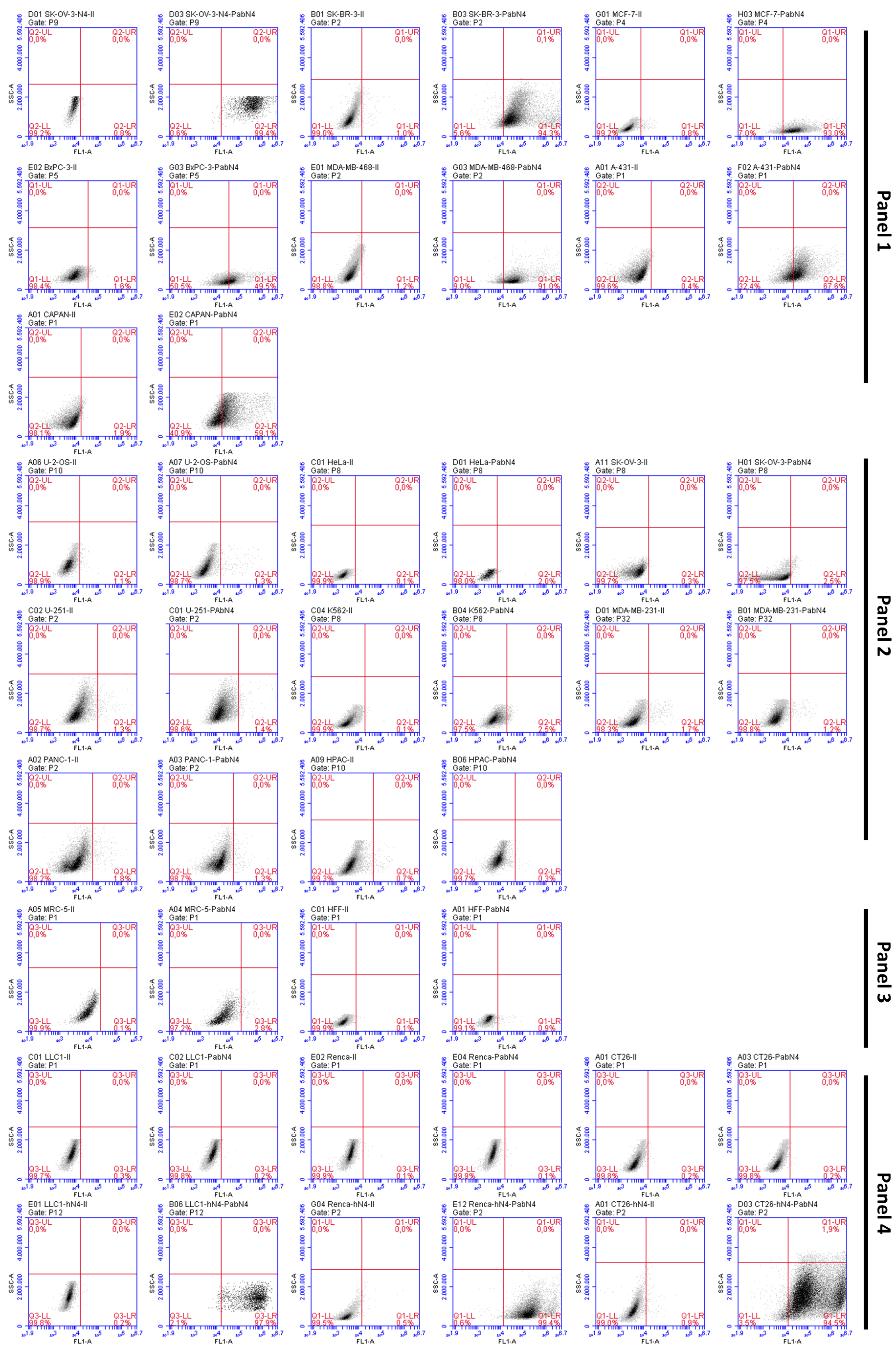


**Supplementary Figure 1.** Flow cytometry dot plots used to quantify human nectin4 in the indicated cell lines and generate Fig. 2A. Cells were reacted with PAb to nectin4 or isotype control and then with fluorescence-conjugated secondary antibody. Cells were gated for cell size on a side scatter (SSC) vs forward scatter (FSC) plot in the range of 2,000,000-7,000,000 (FSC) and 0-1,000,000 (SSC) range (this gate was adjusted for each cell line). To determine positivity to nectin4, gated cells were plotted in SSC vs FL1 (channel for the secondary antibody) dot plot graphs and reported in this figure. Cells reacted with isotype control and secondary antibody were used to determine the nectin4-negative (Q-LL) and -positive (Q-LR) regions (these regions were adjusted for each cell line). Specifically, the nectin4-positive region was set to contain a maximum of 2% of the cells in the samples stained with the isotype control and secondary antibody. The percentage of nectin4-negative and -positive cells is shown in each graph within the corresponding region. For each dot plot graph, median fluorescence intensity values (MeFI) of FL1 were calculated on the entire cell population. MeFI values of the isotype control cells were subtracted from the nectin4-PAb reacted cells and the results were employed to generate Figure 2A.


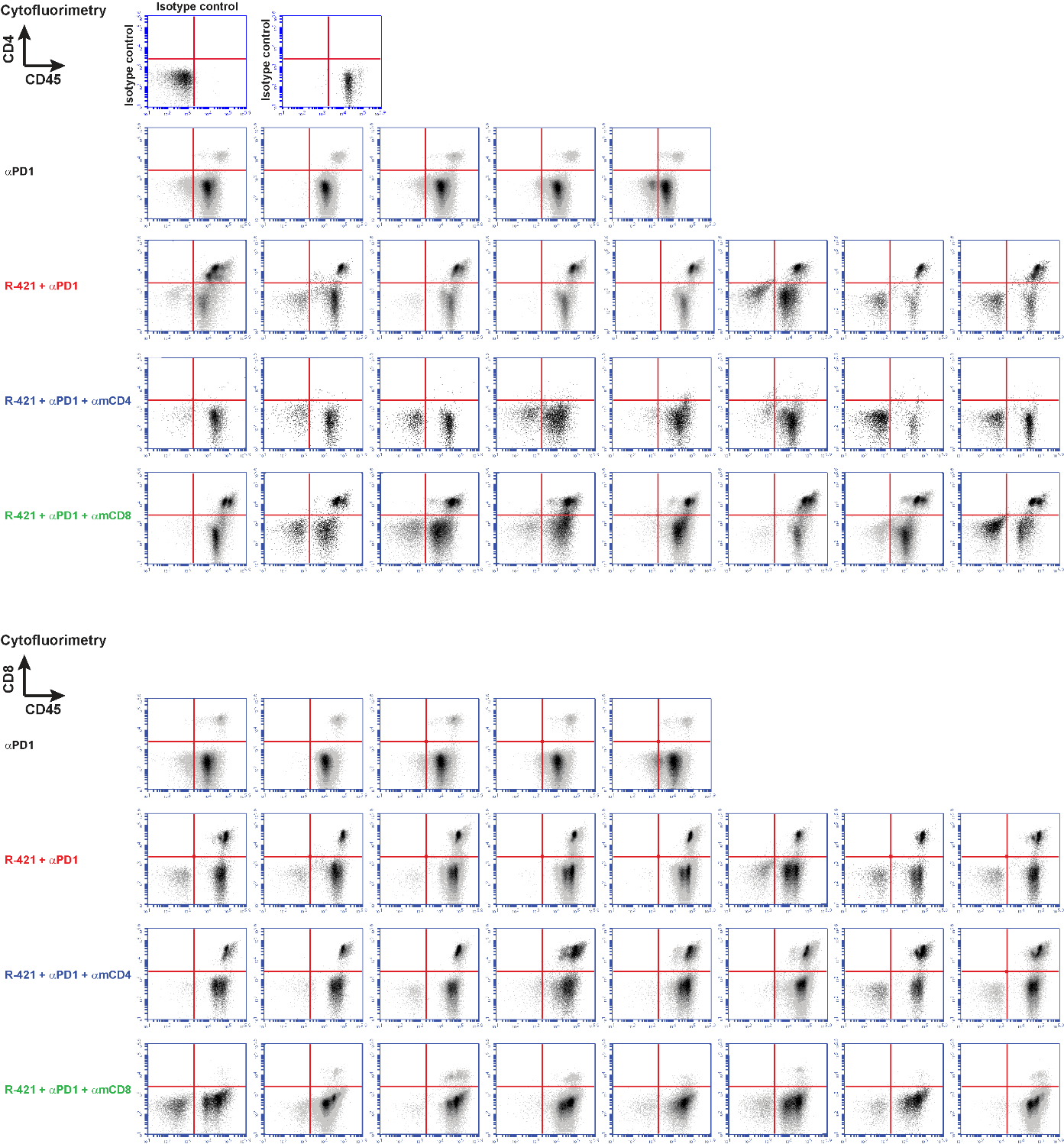


**Supplementary Figure 2.** Flow cytometry dot plots used to determine the amounts of circulating CD4- and CD8-positive cells and generate Figure 9H-I. Cells were reacted with MAb to CD45 (PE-Cy7), MAb to CD4 (FITC), and MAb to CD8 (PE), or the corresponding isotype controls. Cells were gated for cell size on an SSC vs FSC plot in the range of 1,000,000-5,000,000 (FSC) and 0-600,000 (SSC). To determine the percentage of CD4-positive cells within the CD45-positive population, gated cells were plotted in CD4 vs CD45 dot plots reported in this figure. Cells reacted with isotype controls or single staining with MAb for CD45 were used to select the plot areas for CD45^+^CD4^+^ (upper right quadrant, with fluorescence values greater than 2,000 for CD45 and greater than 3,000 for CD4) and CD45^+^ populations (the sum of the upper and lower right quadrants, with fluorescence values greater than 2,000 for CD45). The percentage of CD45^+^CD4^+^ cells within the CD45-positive population is reported in Fig. 9H. The same approach was used to determine the percentage of CD8-positive cells within the CD45-positive population (fluorescence values greater than 3,000 for CD8), and the results are shown in Figure 9I.
